# Transcriptome Analysis Identifies Strategies Targeting Immune Response-Related Pathways to Control Enterotoxigenic *Escherichia coli* Infection in Porcine Intestinal Epithelial Cells

**DOI:** 10.3389/fvets.2021.677897

**Published:** 2021-08-10

**Authors:** Qiong Wu, Defeng Cui, Xinyu Chao, Peng Chen, Jiaxuan Liu, Yiding Wang, Tongjian Su, Meng Li, Ruyu Xu, Yaohong Zhu, Yonghong Zhang

**Affiliations:** ^1^Department of Animal Medicine, College of Animal Science and Technology, Beijing University of Agriculture, Beijing, China; ^2^Beijing Key Laboratory of Traditional Chinese Veterinary Medicine, Beijing University of Agriculture, Beijing, China; ^3^Department of Clinical Veterinary Medicine, College of Veterinary Medicine, China Agricultural University, Beijing, China

**Keywords:** transcriptome (RNA-seq), intestinal epithelia cell, immune response, *Escherichia coli*, porcine, enrichment analysis

## Abstract

Enterotoxigenic *Escherichia coli* (ETEC) is an important cause of post-weaning diarrhea (PWD) worldwide, resulting in huge economic losses to the swine industry worldwide. In this study, to understand the pathogenesis, the transcriptomic analysis was performed to explore the biological processes (BP) in porcine intestinal epithelial J2 cells infected with an emerging ETEC strain isolated from weaned pigs with diarrhea. Under the criteria of |fold change| (FC) ≥ 2 and *P* < 0.05 with false discovery rate < 0.05, a total of 131 referenced and 19 novel differentially expressed genes (DEGs) were identified after ETEC infection, including 96 upregulated DEGs and 54 downregulated DEGs. The Gene Ontology (GO) analysis of DEGs showed that ETEC evoked BP specifically involved in response to lipopolysaccharide (LPS) and negative regulation of intracellular signal transduction. The Kyoto Encyclopedia of Genes and Genomes (KEGG) pathway analysis revealed that immune response-related pathways were mainly enriched in J2 cells after ETEC infection, in which tumor necrosis factor (TNF), interleukin 17, and mitogen-activated protein kinase (MAPK) signaling pathways possessed the highest rich factor, followed by nucleotide-binding and oligomerization domain-like receptor (NLRs), C-type lectin receptor (CLR), cytokine–cytokine receptor interaction, and Toll-like receptor (TLR), and nuclear factor kappa-B (NF-κB) signaling pathways. Furthermore, 30 of 131 referenced DEGs, especially the nuclear transcription factor AP-1 and NF-κB, participate in the immune response to infection through an integral signal cascade and can be target molecules for prevention and control of enteric ETEC infection by probiotic *Lactobacillus reuteri*. Our data provide a comprehensive insight into the immune response of porcine intestinal epithelial cells (IECs) to ETEC infection and advance the identification of targets for prevention and control of ETEC-related PWD.

## Introduction

Enterotoxigenic *Escherichia coli* (ETEC) is one of the most common causes of diarrhea in animals and humans. Enterotoxigenic *Escherichia coli* is responsible for an estimated 75 million diarrheal episodes and approximately 50,000 deaths in children younger than 5 years annually (1). The breakout of ETEC infection in farm animals often attracts a lot of attention due to the possibility of spreading to humans through animal productions. In neonatal and weaned pigs, diarrhea associated with ETEC results in huge economic losses in the pig industry because of high morbidity, mortality, and growth retardation (2, 3). Enterotoxigenic *Escherichia coli*-expressing F4 fimbriae are the most prevalent strains in pigs (4) and include three fimbrial variants of F4ab, F4ac, and F4ad (5). F4ab and F4ac variants are typically associated with diarrhea in pigs (6). The incidence of ETEC infection has become a more frequent reason for severe diarrhea in global swine production. Across Europe, ETEC is detected in 60% of post-weaning diarrhea (PWD)-affected farms (7). Enterotoxigenic *Escherichia coli* is also responsible for the spread of antibiotic resistance in the environment (8). Thus, clarifying the pathogenesis of ETEC is essential for identifying effective prevention strategies for ETEC-related pig diarrhea.

Intestinal epithelial cells (IECs) are in the front line of host defense against pathogens and possess as the crucial mediators of barrier function. Enterotoxigenic *Escherichia coli* interacts with IECs and secretes heat-labile (LT) or heat-stable enterotoxin (ST) enterotoxins, leading to acute intestinal inflammation and diarrhea (9). Besides, IECs are also indispensable for activating innate immune responses and subsequently for inducing adaptive immunity (10). Lipopolysaccharide (LPS) is generally the most potent immunostimulant from Gram-negative bacteria. In general, the innate immune response of IECs is initiated by bind of the pathogen-associated molecular pattern to specialized pattern recognition receptors, including membrane-bound Toll-like receptors (TLRs) and cytoplasmic nucleotide-binding and oligomerization domain-like receptors (NLRs) (11). The detection of pathogen-associated molecular patterns by pattern recognition receptors activates nuclear factor kappa-B (NF-κB) and mitogen-activated protein kinase (MAPK) signaling pathways and triggers transcriptional expression of various pro-inflammatory chemokines, cytokines, and antimicrobial peptides for recruitment and activation of inflammatory cells, thereby inducing host immune responses (12, 13). However, the immune response-related pathogenesis of ETEC, especially the ETEC clinical isolates, is still unclear.

Transcriptome analysis is a useful approach to reveal the comprehensive expression profile of genes involving the host response to pathogen infection. The transcriptomic response of porcine IECs to ETEC reference strains was previously reported. Comparative transcriptomic analysis observed strong differential responses of porcine IECs to F4ab, F4ac, or F18ac ETEC and confirmed that the apical membrane of the IECs represents a first barrier against enteric pathogen infection (9, 14). Enterotoxigenic *Escherichia coli* K88 (serotype O149:K91:K88ac) challenge induces differential expression of genes encoding intestinal ion transporters and water channel aquaporins in young piglets, probably by regulation of the cyclic adenosine monophosphate–protein kinase A signaling pathway (15). The differential genes confirmed by transcriptome analysis can be a potential target for preventing pathogen infection. For example, a soluble and highly fermentable dietary fiber with carbohydrases improves growth performance in pigs infected with F18 ETEC, in part due to a reduction in inflammatory intermediates (16). Dietary supplementation with live yeast limits the early activation of the gene sets related to the impairment of the jejunal mucosa, thus counteracting the detrimental effect of F4 ETEC infection in susceptible pigs (17).

This study aimed to explore the transcriptional change profile of porcine intestinal epithelial J2 cells after infection with a pig diarrhea-related ETEC isolate and determine the potential target for prevention and control for ETEC infection.

## Materials and Methods

### Bacterial Strains

Enterotoxigenic *Escherichia coli* BUA2008 was isolated from the intestinal contents of weaned pigs with diarrhea in our laboratory and used in this study. The ETEC BUA2008 harbors the genes encoding virulence factor *E. coli*-secreted protein A, STa, STb, and LTa.

*Lactobacillus reuteri* BP325 was originally isolated from the gastrointestinal tract of a healthy weaning piglet in our laboratory. The strain was identified through standard morphological, biochemical, and physiological tests and by 16s rRNA gene sequence analysis. *Lactobacillus reuteri* BP325 could inhibit the growth of ETEC BUA2008 and be tolerant to acid and bile salt *in vitro*. *Lactobacillus reuteri* BP325 was grown in De Man, Rogosa, and Sharpe broth (Oxoid, Hampshire, United Kingdom) for 24 h at 37°C under microaerophilic conditions. After overnight incubation, bacteria were inoculated 1:100 in fresh De Man, Rogosa, and Sharpe broth and grown for approximately 8 h until reaching the mid-log phase.

### Intestinal Epithelial Cell Line J2 Cell Culture Condition and Treatment

The porcine intestinal epithelial cell line (IPEC)-J2 cells were cultured in Dulbecco's modified Eagle medium/F12 medium (Invitrogen, Carlsbad, CA, USA) supplemented with 10% heat-inactivated fetal calf serum, 100 μg/ml of streptomycin, and 100 U/ml of penicillin at 37°C in an atmosphere of 5% carbon dioxide and 95% air at 95% relative humidity. Cells (1 × 10^5^ cells/well) were seeded onto a six-well collagen-coated polytetrafluoroethylene transwell filter, and confluent cell monolayers were treated under two different conditions: (i) medium alone (CN); (ii) ETEC infection alone [3 × 10^6^ colony-forming unit (CFU)] at a multiplicity of infection of 30:1 (EC). At 2 h after ETEC infection, the IPEC-J2 cells were washed three times with phosphate-buffered saline (PBS), and then, total RNA was extracted using TRIzol (Invitrogen) according to the manufacturer's instructions for subsequent library construction and sequencing.

For validating whether the genes related to differential inflammatory pathways obtained from RNA-seq were the targets in controlling ETEC infection, confluent J2 cell monolayers were treated under four different conditions: (i) medium alone; (ii) ETEC infection alone (3 × 10^6^ CFU); (iii) incubation with BP325 (3 × 10^7^ CFU) only for 2 h; or (iv) preincubation with BP325 (3 × 10^7^ CFU) for 2 h before exposure to ETEC infection. At 2 h after preincubation with BP325, the IPEC-J2 cells were washed three times with PBS and immediately infected with ETEC (3 × 10^6^ CFU) for 2 h. After infection, the IPEC-J2 cells were washed three times with PBS, and then, total RNA was harvested using TRIzol for quantitative real-time polymerase chain reaction (qRT-PCR) analysis, and the supernatant samples were collected for enzyme-linked immunosorbent assay (ELISA) analysis.

### Library Construction and Sequencing

The RNA integrity was tested by 1% agarose gel electrophoresis, and RNA quality and quantity were evaluated using NanoDrop 2000. The RNA integrity number was further assessed with Agilent 2100 using an RNA 6000 Nano kit (Agilent Technologies, Santa Clara, CA, USA). Samples with qualified purity (RNA integrity number ≥9, OD260/280 ≥1.9, and OD260/230 ≥1.5) were used for subsequent sequencing library preparation. Poly-(A)-containing messenger RNA (mRNA) was enriched using oligo (dT) beads and were broken into short fragments using mentation buffer. The first-strand complementary DNA was synthesized with reverse transcriptase and random hexamer primers using mRNA as templates. Then, the second-strand complementary DNA was synthesized using the buffer, DNA polymerase I, deoxynucleoside triphosphates, and RNase H, followed by end-repair and adapter ligation. Finally, PCR amplification was carried out to obtain the final libraries. The constructed library was quantified and pooled in the flow cell. After cBot clustering using a cBotCluster Generation System, the library preparations were sequenced using Illumina high-throughput sequencing platform Novaseq 6000, and paired-end reads of 150-bp length were generated.

### Genome Alignment and Gene Annotation

The raw reads were quality filtered using Trimmomatic software. The clean reads were obtained after discarding reads containing adapter sequences, low-quality reads with Q10 <30, reads with undetermined base information >10%, and reads that were shorter than 50 bp after the adapter was removed and truncated. The clean reads were aligned to the reference genome Sscrofa11 of the pig (http://asia.ensembl.org/Sus_scrofa/Info/Index) using TopHat v2.1.1 (http://ccb.jhu.edu/software/tophat/index.shtml). The mapped reads were assembled into transcripts using StringTie v1.3.3b (http://ccb.jhu.edu/software/stringtie/). The genes were annotated by BLAST based on Gene Ontology (GO), Cluster of Orthologous Groups of proteins (COG), and Kyoto Encyclopedia of Genes and Genomes (KEGG) databases.

### Analysis of Gene Expression Levels and Identification of Differentially Expressed Genes

The expression of genes was calculated and normalized to fragments per kilobases per million reads (FPKMs) using RSEM v1.3.1 (http://deweylab.biostat.wisc.edu/rsem/). Differentially expressed genes (DEGs) between CN and EC groups were identified using DESeq2 v1.24.0 (http://bioconductor.org/packages/stats/bioc/DESeq2/). The *P*-value was adjusted using Benjamini and Hochberg's approach (BH) for controlling the false discovery rate. Genes with an adjusted *P* < 0.05 and fold change (FC) ≥ 2 or ≤ −2 were assigned as DEGs. A heatmap of DEGs was constructed using the heatmap.2 implemented in the R package gplots (https://cran.r-project.org/web/packages/gplots).

### Gene Ontology and Kyoto Encyclopedia of Genes and Genomes Enrichment Analysis of Differentially Expressed Genes

Gene Ontology enrichment analysis was carried out to specify the potential roles of DEGs based on Fisher's exact test using Goatools v0.6.5 (https://github.com/tanghaibao/GOatools). The *P*-value was adjusted by BH, and GO terms with adjusted *P* < 0.05 were considered significantly enriched. Furthermore, KEGG enrichment analysis was carried out to assess significantly enriched metabolic or signal transduction pathways using KOBAS v2.1.1 (http://kobas.cbi.pku.edu.cn/download.php).

### Venn, Correlation Analysis of Expression, and Protein–Protein Interaction Analysis

A Venn diagram was generated using the Venndiagram R package (https://cran.r-project.org/web/packages/Venndiagram) to obtain EC-unique genes and an overview of the DEGs among different annotation levels. Correlation analysis of gene expression was performed, and differential correlation was calculated using Fisher's transformation of Spearman rank correlation to determine the significance of a change in correlation between two conditions. The statistic *P*-value was corrected by the BH method. Correlations with an absolute Spearman's correlation >0.8 and adjusted *P* < 0.05 were transformed into links between two DEGs, and correlation networks were displayed with Cytoscape v2.8.2. Protein–protein interaction (PPI) analysis of DEGs was carried out based on the Search Tool for the Retrieval of Interacting Genes/Proteins database v11.0 (http://string-db.org/), and the minimum Search Tool for the Retrieval of Interacting Genes/Proteins score was set at 1,000. The interaction with a combined score >0.4 was considered to be significant. The protein network was visualized using Cytoscape v2.8.2.

### Quantitative Real-Time Polymerase Chain Reaction

Quantitative real-time polymerase chain reaction was performed using a QuantStudio 3 RT-PCR system (ThermoFisher Scientific, Waltham, MA, USA). Primer sequences are listed in Supplementary Table 1. Complementary DNA was synthesized and amplified with SYBR Premix DimerEraser (TakaRa Biotechnology Inc., Shiga, Japan). Each sample was assayed in duplicate. Relative quantification of mRNA expression was performed by normalizing the cycle threshold values of the target genes to the cycle threshold values of the housekeeping gene encoding. The 2^−Δ*ΔCT*^ method relative to the reference gene encoding glyceraldehyde-3-phosphate dehydrogenase was used to analyze the FC of target genes between CN and EC groups.

### Enzyme-Linked Immunosorbent Assay

The concentrations of interleukin (IL)-1β and IL-6 were determined using commercially available porcine-specific ELISA kits (IL-1β: #PLB00B, IL-6: #P6000B; R&D Systems, Minneapolis, MN, USA) according to the manufacturer's instructions.

### Data Accession Number

The raw transcriptome data have been deposited in the US National Center for Biotechnology Information Sequence Read Archive database under accession no. SRR13291685–SRR13291693.

### Statistical Analysis

Statistical analysis was performed using the IBM SPSS Statistics 21 (SPSS Inc., Chicago, IL, USA) software package. Natural logarithm transformation was performed before analysis for ELISA data of IL-1β and IL-6 to achieve a normal distribution. Differences between means were compared using one-way analysis of variance, followed by Tukey's honestly significant difference *post-hoc* test. A *P*-value of < 0.05 was considered statistically significant. Data were visualized using GraphPad Prism 5 software (Graphpad Software Inc., San Diego, CA, USA). All experiments were performed three times.

## Results

### Quality Control and Transcriptome Assembly

A total of 348.2 million raw reads (approximately 52.6 Gbps) were yielded across six samples. After removing low-quality reads, ambiguous and adaptor reads, retaining 344 million high-quality clean reads (approximately 51.2 Gbps, 50.6–61.7 million reads for each sample) were used for subsequent assembly and analysis. The average of Q20 and Q30 of clean reads across all samples was above 98.3 and 94.9%, respectively, indicating that the obtained clean reads were of high quality. Compared with the pig reference genome Sscrofa11, 93.5 and 93.2% of total reads for CN and EC groups were uniquely mapped on the *sus scrofa* genome, respectively, and the GC content for CN and EC groups was more than 51.2% (Supplementary Table 2).

**Figure 1:**
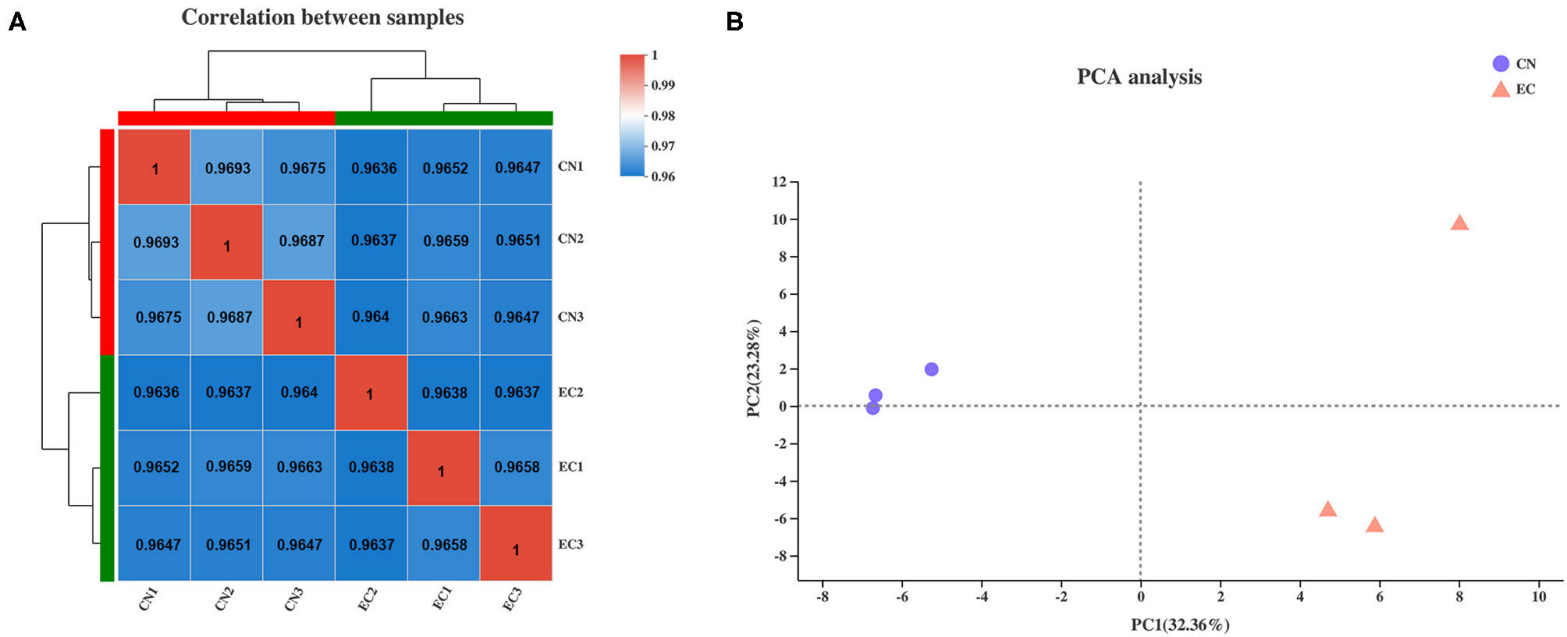
Correlation analysis and principal component analysis of samples. **(A)** Heatmap of hierarchical clustering for six samples using Spearman method. Bold values represented *R*^2^ for replicates of each sample. **(B)** Principal component analysis plot of samples. Each point represented one sample. Percentages were contribution ratios.

The clean reads of genes were normalized to FPKM values to precisely evaluate the gene expression levels. The saturation curve of sequencing showed that the FPKM values of approximately 42% of genes in CN and EC groups were below 3.5, and only a few of 7% genes were highly expressed with FPKM values higher than 60. Most of the genes with medium or above expression levels (i.e., the genes with FPKM value of 3.5–60) were nearly saturated at 51% of the sequencing reads (ordinate value tended to 1), indicating that the overall quality of saturation was high, and the sequencing quantity could cover most of the expressed genes (Supplementary Table 3 and Supplementary Figure 1). A Spearman's correlation matrix analysis of the FPKM distribution among biological replicates for all six samples showed that the correlation indices of the mapped genes in different groups were different, but the differences within the groups were small, indicating a high consistency of measurements within each group and high reproducibility of the sequencing data (Figure 1A). Principal component analysis was carried out to evaluate the clustering nature of these samples. Principal components 1 and 2 explained 32.4 and 23.3% of the distributions of the different groups, respectively (Figure 1B). The samples of each group were clustered together, and data showed good correlation and repeatability.

### Analysis of Gene Expression and Functional Annotation

**Figure 2:**
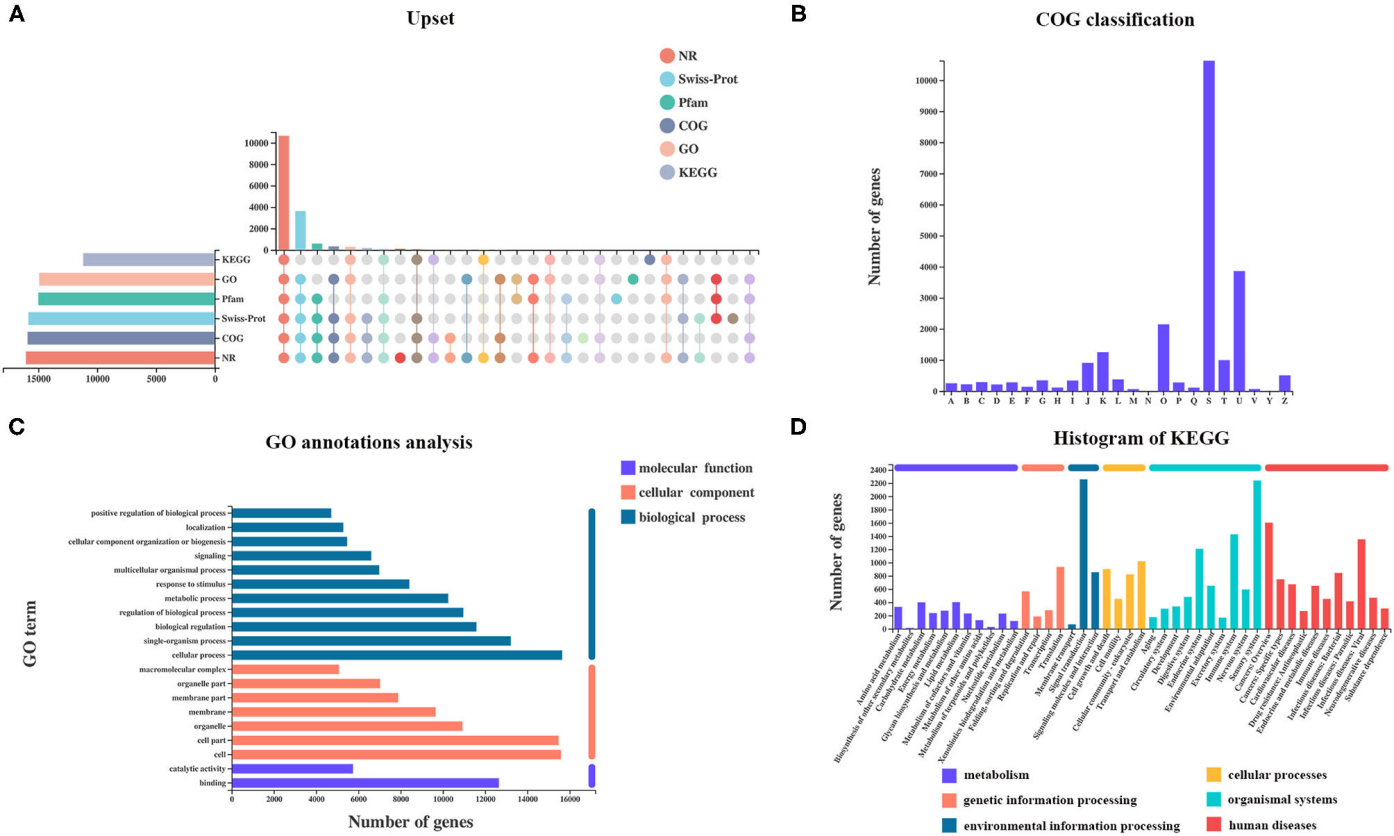
Analysis of referenced gene expression and functional annotation. **(A)** Number of genes annotated using KEGG, GO, Pfam, Swiss-Prot, COG, and NR datasets. **(B)** COG, **(C)** GO, and **(D)** KEGG classifications of referenced genes were summarized.

A total of 27,001 genes were found from all samples, including 25,880 (95.8%) referenced genes and 1,121 (4.2%) unannotated novel genes. Of 25,880 referenced genes, a total of 23,375 genes could be annotated using GO (20,488), KEGG (16,308), COG (22,389), NR (23,133), Swiss-Prot (22,155), and Pfam (20,554) databases (Figure 2A). The COG database contains orthologous proteins and is used to classify and predict their possible function. The referenced genes were assigned into 22 orthologous COG clusters (Figure 2B). Some genes may be assigned into several clusters in COG categories, whereas some were assigned to the same cluster but with different protein orthologous similarities. A total of 10,628 genes were assigned to “unknown function” (class S). The majority of genes were distributed in “intracellular trafficking, secretion, and vesicular transport” (class U; 3,861 genes), followed by “post-translational modification, protein turnover, chaperones” (class O; 2,149 genes), and “transcription” (class K; 1,252 genes).

Gene Ontology analysis revealed that a total of 20,481 genes were mapped to 64 GO terms, with 17,454 genes assigned to biological process (BP), 18,2023 genes assigned to cellular component (CC), and 17,580 genes assigned to molecular function (MF). In the BP ontology, cellular processes (15,647 genes) and single-organism processes (13,216 genes) were the most enriched GO terms. In the CC ontology, the most enriched terms were cell (15,592 genes), and cell part (15,476 genes) was the most enriched GO term. In the MF ontology, binding (12,651 genes) and catalytic activities (5,743 genes) were the most enriched GO terms (Figure 2C).

Kyoto Encyclopedia of Genes and Genomes pathway analysis helps to better understand the biological functions of genes. A total of 3,209 genes were annotated and classified into six first KEGG categories (metabolism, environmental information processing, cellular processes, genetic information processing, human diseases, and organismal system) and 335 second KEGG categories. Signal transduction (2,257 genes) was the most abundant KEGG pathway, followed by the sensory system (2,240 genes) (Figure 2D).

A total of 12,227 novel transcripts were found in the 61,408 transcripts identified, including 389 transcripts with exonic overlap with reference on the opposite strand, 506 with transfer falling entirely within a reference intron, 9,450 with potentially novel isoform: at least one splice junction was shared with a reference transcript, 1,265 unknown and intergenic transcripts, and 350 with generic exonic overlap with a reference transcript (Supplementary Figure 2).

A total of 1,106 novel genes (accounting for 98.7% of all novel genes) and 11,670 novel transcripts (accounting for 95.4% of all novel transcripts) were successfully annotated after BLAST, with 242 and 8,280 in the GO database, 63 and 6,932 in the KEGG database, 289 and 9,906 in COG database, 757 and 10,909 in the NR database, 187 and 9,782 in the Swiss-Prot database, and 122 and 8,907 in the Pfam database, respectively (Supplementary Figure 3A, Supplementary Table 4). The COG analysis showed that 307 novel genes were assigned into nine COG functional categories (Supplementary Figure 3B), including “chromatin structure and dynamics” (class B; 21 genes), “post-translational modification, protein turnover, chaperones” (class O; 11 genes), “intracellular trafficking, secretion, and vesicular transport” (class U; 7 genes), “translation, ribosomal structure, and biogenesis” (class J; 6 genes), “energy production and conversion” (class C; 4 genes), “transcription” (class K; 3 genes), “signal transduction mechanisms” (class T; 3 genes), and “cytoskeleton” (class Z; 2 genes). Importantly, 814 genes not assigned to any COG class, as well as 250 genes of unknown function (class S), were enriched in the novel genes. According to the GO analysis, the novel genes were divided into multiple functional groups (Supplementary Figure 3C), in which MF, BP, and CC were the most enriched terms. Catalytic activity (131 genes) and binding (96 genes) in MF, membrane (108 genes) and membrane part (104 genes) in a CC, and cellular process (87 genes) and metabolic process (76 genes) in the BP were the most enriched ontology terms. A total of 40 novel genes were classified into 112 KEGG pathways, mainly functioning in the phagosome, Ras signaling pathway, cyclic adenosine monophosphate signaling pathway, calcium signaling pathway, endocytosis, and tuberculosis (Supplementary Figure 3D).

### Identification of EC-Unique Genes and Functional Analysis

**Figure 3:**
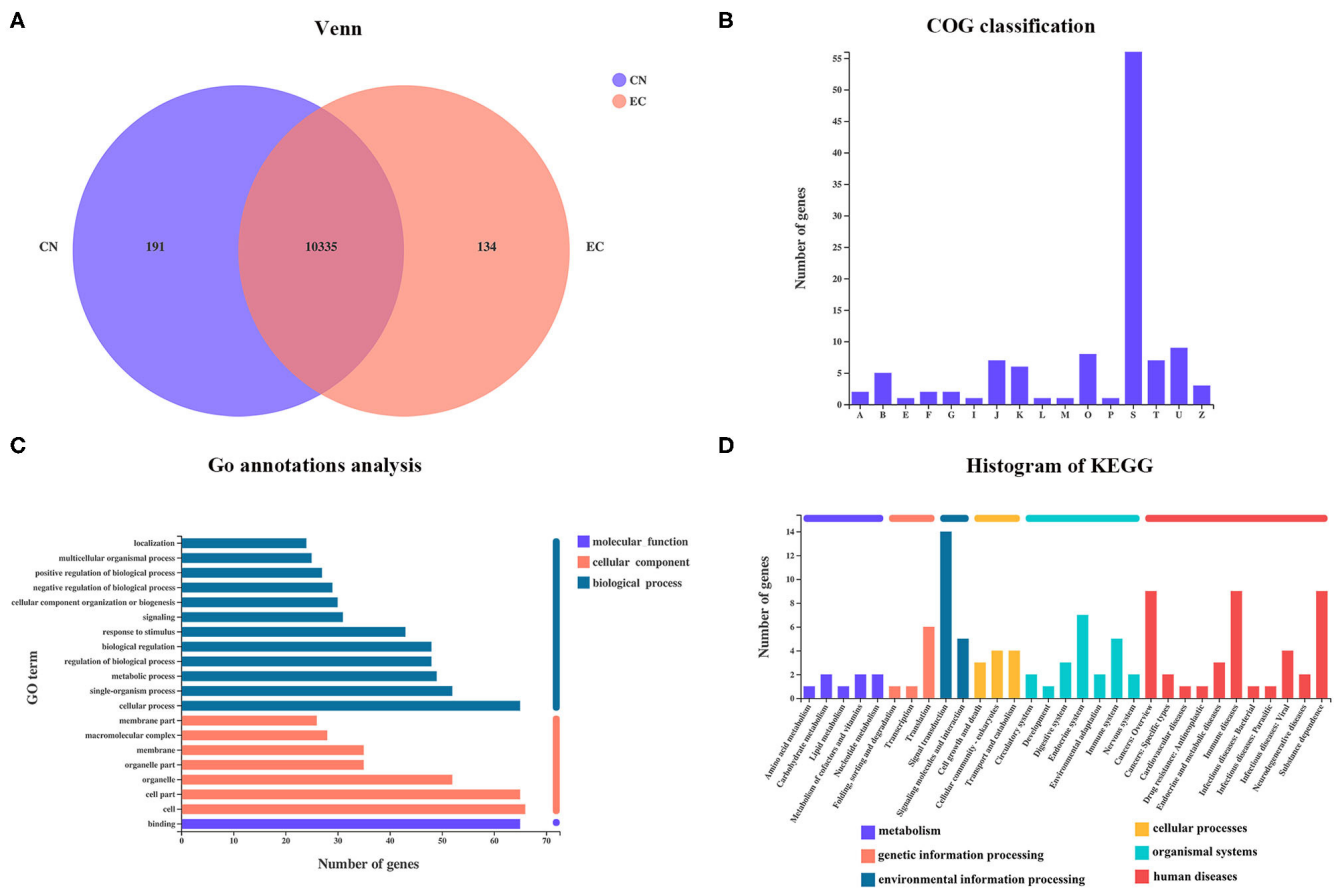
Identification of EC-unique genes and functional analysis. **(A)** Venn diagram of shared referenced genes between CN and EC groups. **(B)** COG, **(C)** GO, and **(D)** KEGG classifications of 134 EC-unique genes were summarized.

From the Venn diagram, 134 referenced genes were uniquely present in the EC group (Figure 3A). A total of 55 EC-unique genes could be annotated to 15 COG functional categories, including “intracellular trafficking, secretion, and vesicular transport” (class U; 9 genes), “post-translational modification, protein turnover, chaperones” (class O; 8 genes), “translation, ribosomal structure, and biogenesis” (Class J; 7 genes), “signal transduction mechanisms” (class T; 7 genes), “transcription” (class K; 6 genes), and so on (Figure 3B). According to the GO analysis, 96 of 134 EC-unique genes were assigned to 45 GO terms and were specific to the cellular process, cell, cell parts, and binding (Figure 3C). A total of 47 EC-unique genes were assigned to 86 second KEGG categories, mainly functioning in signal transduction, immune diseases, substance dependence, immune system, and translation (Figure 3D).

### Analysis of Differentially Expressed Genes

**Figure 4:**
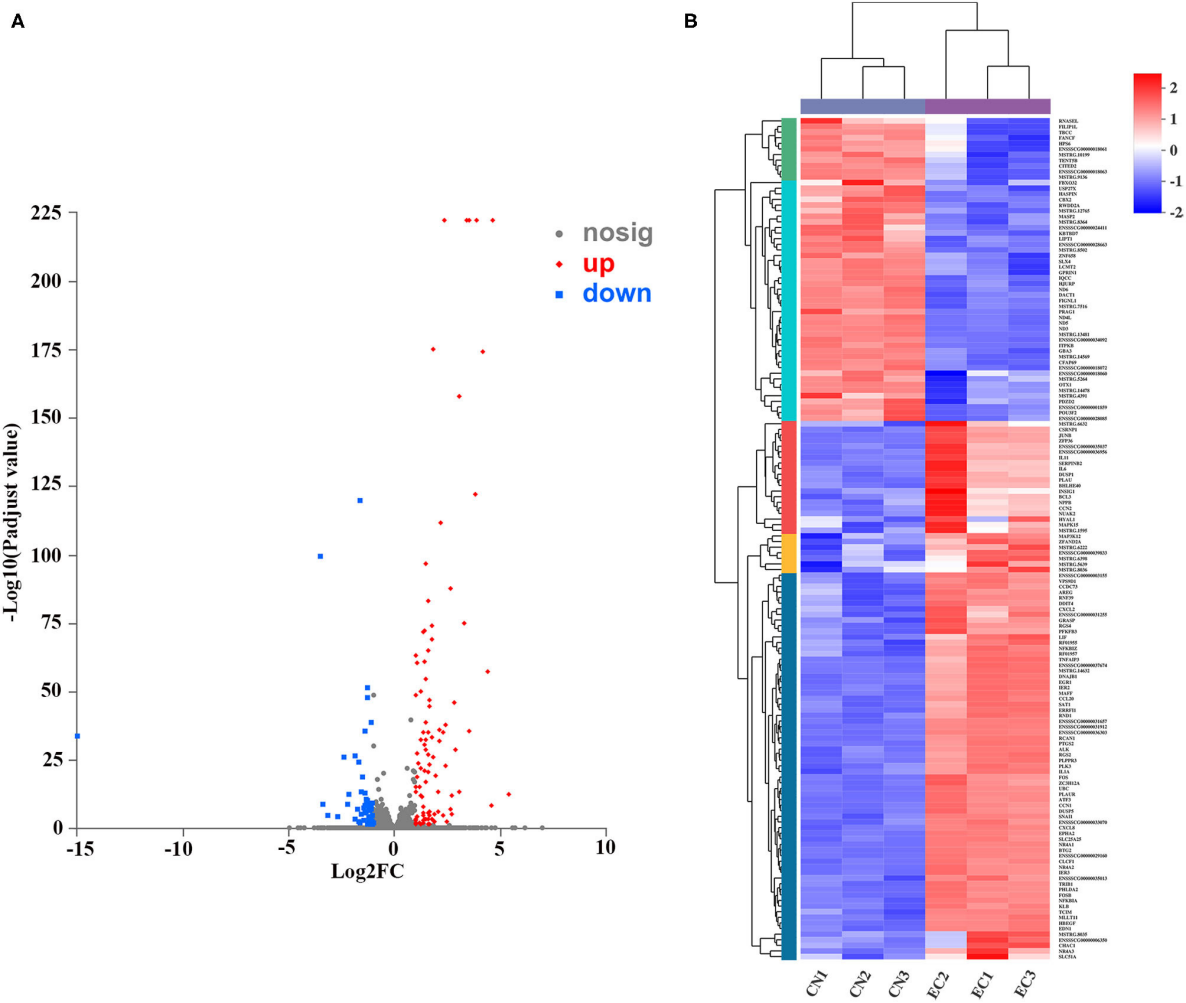
Identification and expression of DEGs. **(A)** Volcano plot of DEGs between CN and EC groups. X-axis represented fold change of expression of DEGs, and Y-axis represented statistical significance of fold change. Each point represented a DEG. Red dots represented significantly upregulated DEGs; blue dots represented significantly downregulated genes; gray dots represented insignificantly DEGs. **(B)** Cluster heatmap of DEGs. Rows and columns represented genes and samples. Legend represented Log_2_FC of gene abundance. Red and blue indicated high and low expression of DEGs, respectively.

A rigorous comparison at adjusted *P* < 0.05 and log_2_FC ≥ 1 for upregulated genes or log_2_FC ≤ −1 for downregulated genes using the DESeq method were carried out to identify the DEGs. The list of DEGs, along with their FCs and annotations, were presented in Supplementary Table 5. A total of 150 DEGs (131 referenced genes and 19 novel genes) between CN and EC groups were found, including 96 upregulated DEGs and 54 downregulated DEGs (Figure 4A). The hierarchical cluster heatmap showed that expression profiles of DEGs after ETEC infection were distinctly different (Figure 4B). The top 10 upregulated genes were *CCL20, NR4A1, NR4A3, FOSB, NR4A2, EGR1, FOS, RCAN1, DDIT4*, and *CXCL2*. The top 10 downregulated genes were *GBA3, POU3F2, CFAP69, MASP2, ND5, USP27X, FIGNL1, CBX2, KBTBD7*, and *LCMT2*.

### Gene Ontology Function Analysis of Differentially Expressed Genes

**Figure 5:**
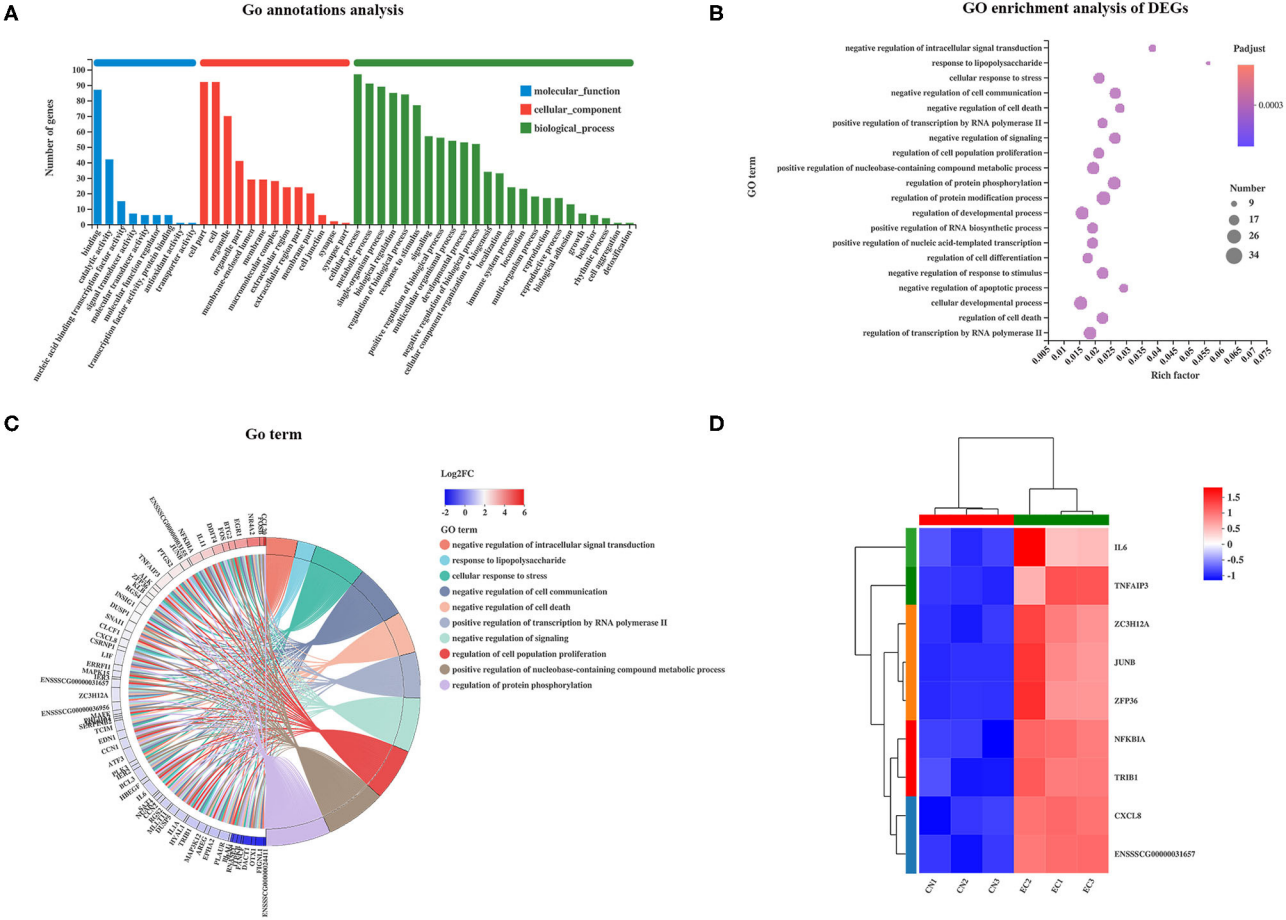
GO enrichment analysis of DEGs. **(A)** GO functional annotation of DEGs. **(B)** GO enrichment analysis of DEGs. Enriched bubble chart showing enrichment of GO functions. X-axis represented enrichment ratio, and Y-axis represented top 20 GO terms. Number: Bubble size represented number of genes annotated to a KEGG Pathway. Padjust: color indicated enriched adjusted *p*-value. **(C)** GOChord plot of top 10 ranked GO terms. Chords indicated a detailed relationship between expression levels of DEGs (left semicircle perimeter) and their enriched GO terms (right semicircle perimeter). Genes are linked to their annotated terms *via* colored ribbons. **(D)** Heatmap of DEGs enriched in GO term “the response to LPS” showing the expression profile.

Gene Ontology analysis was performed to characterize the functional terms of gene expression changes exposure to ETEC infection. Among the 131 referenced DEGs mentioned earlier, 113 were divided into three principal GO terms of level 2 (Figure 5A): MF (101 genes, 9 GO terms of level 2), CC (101 genes, 13 GO terms of level 2), and BP (101 genes, 24 GO terms of level 2). The DEGs assigned to the cellular process (97 genes) occupied the maximum proportion, followed by that assigned to cell (92 genes), cell part (92 genes), and metabolic process (91 genes). The GO enrichment analysis was performed at the level of adjusted *P* < 0.05. The top 20 ranked GO terms of DEGs are shown in Figure 5B. The response to LPS and negative regulation of intracellular signal transduction terms classified into BP class occupied the strongest enrichment degrees. The nine DEGs encoding NF-κB inhibitor alpha, tribbles pseudokinase 1, tumor necrosis factor (TNF) alpha-induced protein 3, C-X-C motif chemokine ligand 8, AP-1 transcription factor subunit JunD proto-oncogene and JunB proto-oncogene, ZFP36 ring finger protein, zinc finger CCCH-type containing 12A, and IL-6 were directly related to response to LPS and were unregulated in the EC group (Figures 5C,D). The regulation of protein modification process term (34 genes) contained the most abundant DEGs, followed by cellular developmental process (32 genes), and regulation of developmental process (31 genes).

### Kyoto Encyclopedia of Genes and Genomes Pathway Analysis of Differentially Expressed Genes

**Figure 6:**
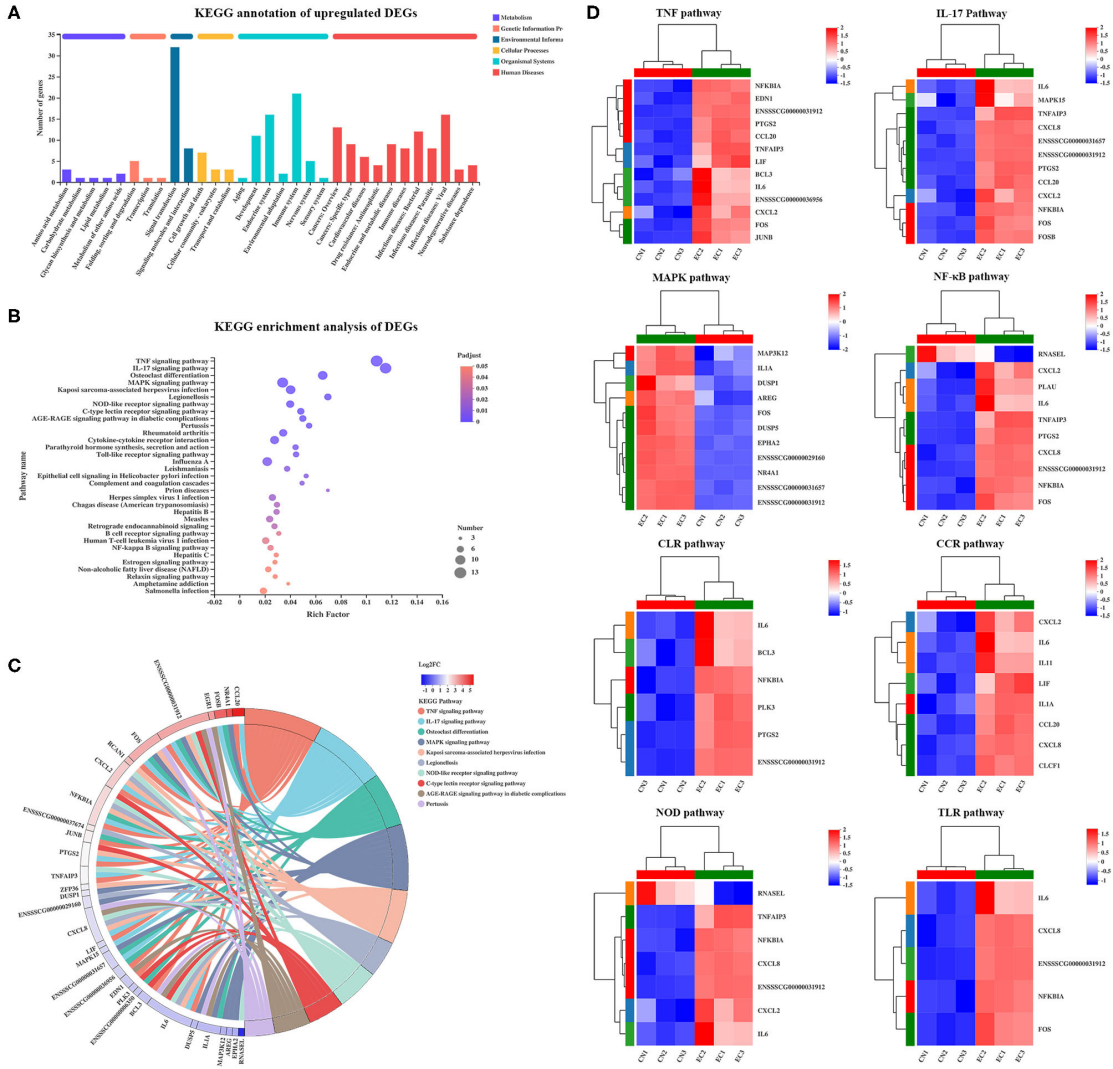
KEGG enrichment analysis of DEGs. **(A)** KEGG pathway annotation of DEGs. **(B)** KEGG enrichment analysis of DEGs. Enriched bubble chart showing enrichment of KEGG pathways. X-axis represented enrichment ratio, and Y-axis represented top 20 KEGG pathways. Number: Bubble size represented number of genes annotated to a KEGG Pathway. Padjust: color indicated enriched adjusted *p*-value. **(C)** KEGGChord plot of top 10 ranked KEGG pathways. Chords indicated a detailed relationship between expression levels of DEGs (left semicircle perimeter) and their enriched KEGG pathways (right semicircle perimeter). Genes are linked to their annotated terms *via* colored ribbons. **(D)** Heatmap of DEGs enriched in immune response-related KEGG pathways showing expression profile in CN and EC groups.

Of 131 referenced DEGs, 55 upregulated and 15 downregulated DEGs were annotated to 152 second KEGG categories. The KEGG pathways enriched in the upregulated DEGs were mainly specific to signal transduction (32 genes) and immune system (21 genes), and the KEGG pathways enriched in the downregulated DEGs were mainly specific to environmental adaptation (four genes) and energy metabolism (four genes) (Figure 6A). Kyoto Encyclopedia of Genes and Genomes Pathway enrichment analysis was performed to identify differential genes-related signal transduction and biochemical metabolic pathways. As a result, a total of 33 KEGG pathways were significantly enriched and were mainly involved in the immune response. Among these pathways, the TNF (adjusted *P*-value of 2.52E^−11^), IL-17 (adjusted *P*-value of 5.29E^−11^), and MAPK (adjusted *P*-value of 9.06E^−5^) signaling pathways possessed the highest rich factor (Figure 6B). Besides, some immune response-associated KEGG pathways were also enriched, including NLR (adjusted *P*-value of 6.61E^−5^), C-type lectin receptor (CLR) (adjusted *P*-value of 2.56E^−5^), cytokine–cytokine receptor interaction (adjusted *P*-value of 2.52E^−11^), TLR (adjusted *P*-value of 4.63E^−4^), and NF-κB (adjusted *P*-value of 6.4E^−3^) signaling pathways. Furthermore, the DEGs mapped to the immune response-associated KEGG pathways mentioned earlier and their expression patterns were identified (Figure 6C). Of the 131 DEGs, a total of 30 DEGs were involved in the immune response-associated KEGG pathways discussed earlier (Figure 6D), including TNF (13 genes), IL-17 (12 genes), MAPK (11 genes), NLR (7 genes), CLR (8 genes), cytokine–cytokine receptor interaction (8 genes), TLR (5 genes), and NF-κB (5 genes) signaling pathways. These 30 “core DEGs” determined the IPEC-J2 cell immune response to ETEC infection.

### Correlation Analysis of Expression of “Core Differentially Expressed Genes” and Protein–Protein Interaction

**Figure 7:**
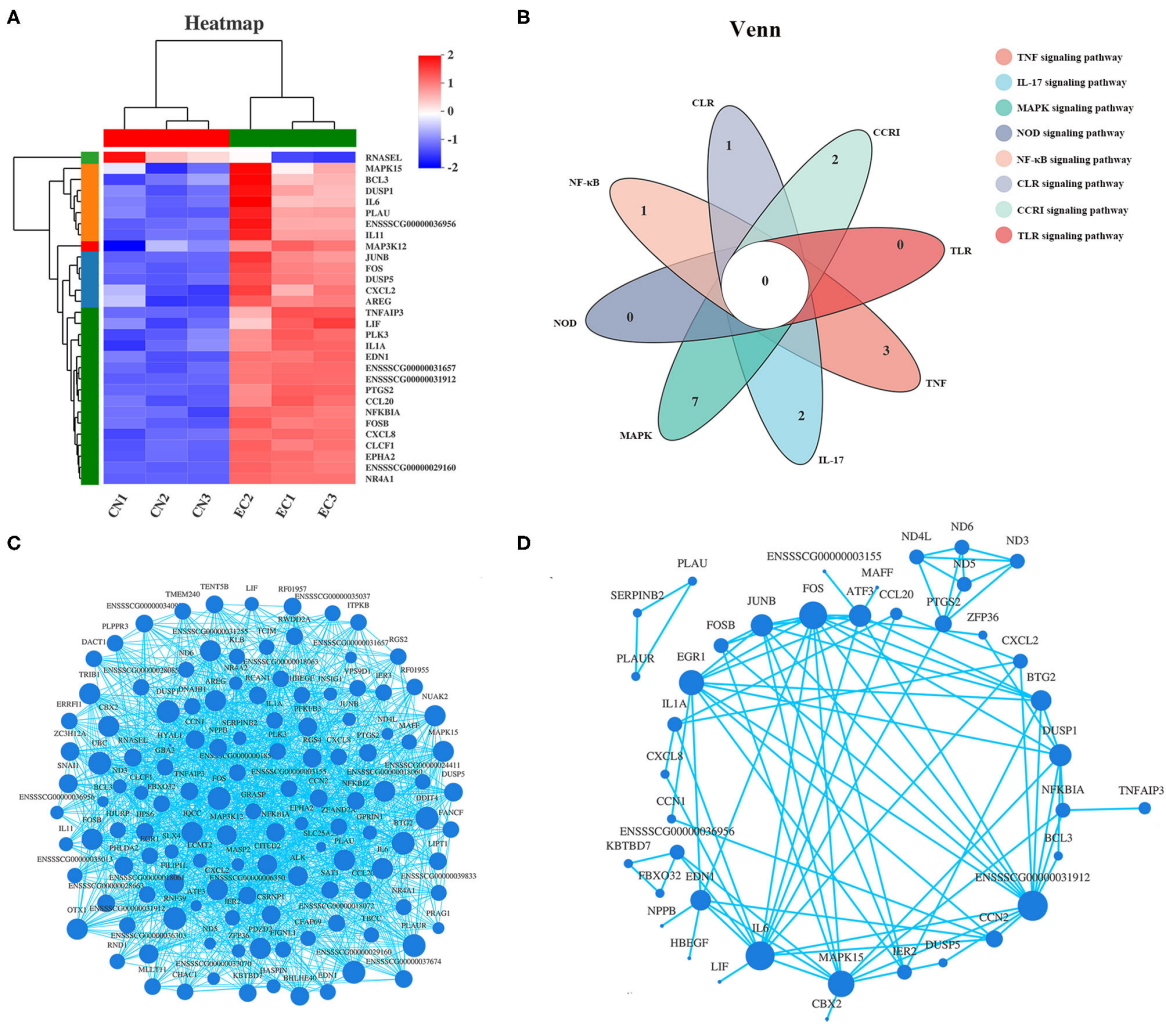
Expression pattern of “core DEGs.” **(A)** Heatmap of “core DEGs” showing expression profile in CN and EC groups. **(B)** Venn diagram of “core DEGs” enriched in immune response-related pathways. **(C)** Correlation analysis and **(D)** protein–protein interaction of gene expression of 131 referenced DEGs providing a rationale for functional significance of “core DEGs.” Size of each node corresponded to number of connections (degree).

Among the 30 “core DEGs,” only the *RNAsel* gene encoding ribonuclease L was downregulated, and the other 29 genes were upregulated after ETEC infection (Figure 7A and Supplementary Table 6). According to the Venn diagram (Figure 7B), some “core DEGs” were directly related to several inflammatory signaling pathways, whereas some were assigned to the specific signaling pathways. For example, two genes (*FOS* and ENSSSCG00000031912) participated in ETEC-induced activation of TNF, IL-17, and MAPK signaling pathways. Five genes (*NFKBIA, FOS, CXCL8, IL-6*, and ENSSSCG00000031912) were involved in the TLR and NF-κB signaling pathways. No gene was related to all inflammatory signaling pathways. Correlation analysis of gene expression in the 131 DEGs provided a rationale for the functional significance of “core DEGs” (Figure 7C). Some genes in the “core DEGs,” such as *MAPK15, IL-*6, *CXCL2, NFKBIA, FOS*, and *TNFAIP3*, had the strongest correlation with other genes, indicating that the “core DEGs” had a coordinating function in the J2 cell response to ETEC infection. Protein products of DEGs among CN and EC groups identified multiple interactions with medium to high confidence (scores ranging from 0.4 to 1). The main protein interaction cluster derived from the 131 DEGs contained 40 nodes, each representing one protein and connected by 89 edges (Figure 7D). The *IL-6, FOS, MAPK15, JUNB, EDN1, DUSP1*, and the gene with unknown function (gene id: ENSSSCG00000031912) had the highest scores for betweenness centrality, indicating they were most important for connections with other proteins.

### Quantitative Real-Time Polymerase Chain Reaction and Enzyme-Linked Immunosorbent Assay Validation of “Core Differentially Expressed Genes”

**Figure 8:**
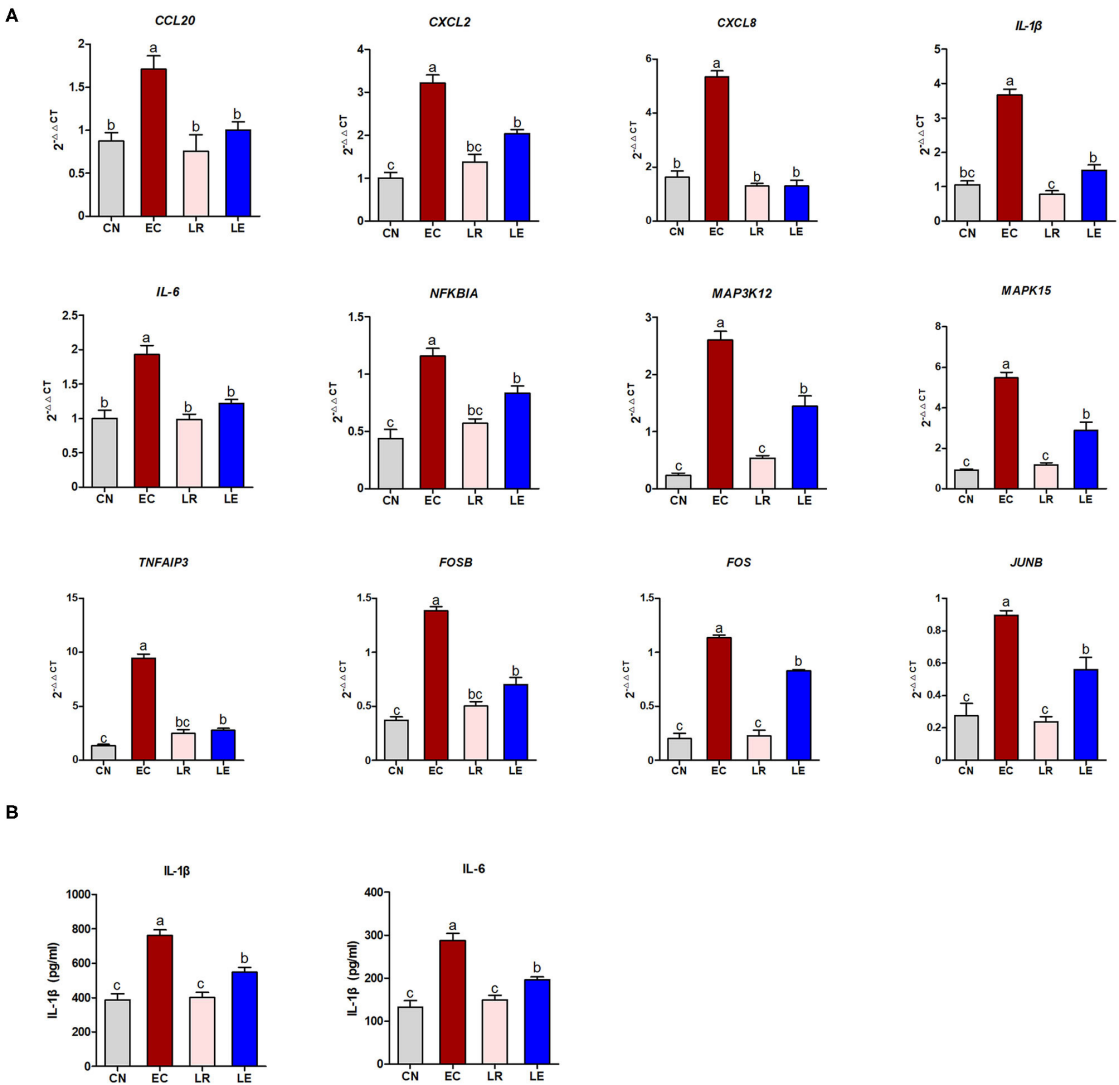
qRT-PCR and ELISA validation of selected DEGs. **(A)** qRT-PCR was performed to validate immune response-related 12 selected DEGs. **(B)** ELISA was performed to measure concentration of IL-1β and IL-6.

According to the KEGG enrichment analysis, correlation analysis of gene expression, and PPI results, 12 “core DEGs” (*CCL20, CXCL2, CXCL8, IL-1*β, *IL-6, NFKBIA, MAP3K12, MAPK15, TNFAIP3, FOSB, FOS*, and *JUNB*) involving the inflammatory signaling pathways were chosen for qRT-PCR to validate the RNA-seq results. The results showed that the differential expression patterns of the 12 “core DEGs” were in accord with those detected by RNA-seq analysis (Figure 8A), indicating that the RNA-seq data could reliably reflect the alteration of gene expression. The operational errors in experimental treatments may result in differences in FC expression.

To further validate whether these “core DEGs” were the targets in controlling ETEC infection, the J2 cells were pre-incubated with probiotic *L. reuteri* BP325, followed by ETEC infection. Figure 8A shows that pretreatment with probiotic *L. reuteri* BP325 significantly attenuated the ETEC-induced increased mRNA expression in the 12 “core DEGs.” Furthermore, pretreatment with probiotic *L. reuteri* BP325 significantly suppressed the elevated IL-1β and IL-6 levels in the supernatant of J2 cells caused by ETEC (Figure 8B).

## Discussion

Enterotoxigenic *Escherichia coli*'s are the most prevalent intestinal pathogen strains causing PWD in pigs (18). Understanding the pathogenic mechanism of ETEC is the primary task of prevention and control of PWD. The criteria set of *P*-value and FC significantly affects the number of DEGs and results of functional and pathway enrichment analysis. A previous study showed that 2,443 DEGs were found in J2 cells infected F4ab ETEC with under the criteria of |FC| > 1.5 and *P* < 0.05 with FDR < 0.252, whereas only 1,188 DEGs were found under the criteria of |FC| > 2 and *P* < 0.05 (9). This study was attempted to identify the main response pathways induced by ETEC as prevention targets in the future, and thus a rigorous comparison at |FC| ≥ 2 and *P* < 0.05 with FDR < 0.05 was carried out. A total of 96 upregulated and 54 downregulated DEGs mainly functioned in response to LPS and negative regulation of intracellular signal transduction terms. Pathway enrichment analysis revealed that the DEGs were mostly enriched in TNF, IL-17, and MAPK signaling pathways, followed by immune response-associated NLR, CLR, cytokine–cytokine receptor interaction, TLR, and NF-κB signaling pathways. Of 131 referenced DEGs, 30 DEGs were involved in the immune response-related pathways discussed earlier, namely “core DEGs,” and could represent the target molecules for the prevention and control of enteric ETEC infection. Correlation analysis of gene expression and PPI showed that IL-6 was located in the center of the protein interaction network, with the most connections with other proteins. Furthermore, the RNA-seq data were validated using qRT-PCR and ELISA methods. Finally, we verified the potential of the DEGs as targets of prevention and control for ETEC infection through pretreating J2 cells with a probiotic *L. reuteri* strain isolated from a healthy swine gut.

By comparing the gene expression level in CN and EC groups to produce a Venn diagram, we found 134 referenced genes uniquely present in the EC group. These EC-unique genes mainly functioned in cellular processes, such as nucleosome assembly, DNA packaging complex, or protein–DNA complex. Nucleosomes serve as the repeating units of cellular chromatin and play an important role in innate immune responses (19). Physiologically, nucleosome assembly is typically associated with DNA replication. It was reported that adenovirus-encoding protein VII interacts with cellular chromatin and binds nucleosomes, leading to sequestration of the high-mobility group protein B family members and abrogation of immune responses (20). These EC-unique gene functions may represent an immune evasion strategy of pathogens in which nucleosome binding is exploited to control the immune response.

Gene Ontology enrichment analysis of 131 referenced DEGs revealed the regulation of protein modification process term (34 genes) contained the most abundant DEGs, whereas the response to LPS and negative regulation of intracellular signal transduction terms classified into BP class occupied the strongest enrichment degrees. Comparable studies have shown that ETEC induced typical immune-related signaling pathways in porcine IECs, but the extent of the inflammatory response is different due to the difference in ETEC strain, infection time, infection concentration, and cell lines. The cell junction organization and extracellular matrix organization terms are enriched in the DEGs of ETEC strain 11501-infected IPEC-1 cells (21).

By KEGG analysis, 33 KEGG pathways enriched were mainly involved in the immune response. Toll-like receptor, NF-κB, and TNF signaling pathways were significantly enriched in the EC group. Toll-like receptor signaling pathway is crucial for the regulation and activation of numerous pro-inflammatory molecules. *Escherichia coli* LPS binding to TLR4 promotes activation of downstream TRIF-dependent and MyD88-dependent pathways, which in turn induces production of a cascade of activated molecules, such as NF-κB, AP-1, and inflammatory cytokines (22). Consistent with the present study, enriched TLR and NF-κB signaling pathways are also found in porcine IECs infected with various ETEC strains (9, 21). NF-κB contains a family of transcription factors, which functions in regulating various biological responses, including proliferation, cell apoptosis, and invasiveness. Phosphorylated IκB (an inhibitor of p65) activates p65 and induces the activation of NF-κB. In our study, *UBC* encoding ubiquitin C and *TNFAIP3* encoding TNF alpha-induced protein 3 were significantly regulated after ETEC infection. Ubiquitin is a highly conserved protein, functioning as a tag in the selective proteolysis of 26S proteasome to abnormal proteins. UBC serves as an intermediary molecule in the activation of NF-κB signaling by TNF (23). UBS could modify many cytosolic proteins recruited by TNF/TNF-R1 and then regulates the activity of the NF-κB pathway. These data indicate that ETEC may modulate the TLR and NF-κB pathways *via* TNF-UBC pathways.

C-type lectin receptor constitutes a large superfamily of proteins that act as pattern-recognition receptors for pathogen-derived carbohydrates (24). C-type lectin receptor pathway is enriched in the IPEC-1 cells infected with ETEC strain 11501 (21). Mitogen-activated protein kinase is a downstream cascade pathway of many growth-factor receptors (25), which is activated by a broad array of extracellular stimuli. Mitogen-activated protein kinase pathway regulates and participates in various BPs, such as immune response to pathogen infection and focal adhesion (26) that also controlling cell communication (27). In this study, *MAP3K12* and *MAPK15* genes were related to the enriched MAPK pathway. *MAP3K12* and *MAPK15* genes are associated with p38 and ERK MAPK signaling pathways, respectively. MAP3K21 is a negative regulator of TLR4 signaling (28). MAP3K21 is strongly correlated with F4ac ETEC-related diarrhea in pigs (14). Activation of p38 MAPK induces phosphorylation of c-Jun and promotes its transactivation activity; in turn, c-Jun combines to target gene promoters as heterodimers, AP-1, with c-Fos (29). ERK MAPK pathway mediates the production of pro-inflammatory cytokine IL-8, IL-1β, and TNF-α *via* TLR2/TLR4 (30).

IL-17 is a pro-inflammatory cytokine family and exerts unique functions to bridge the innate and adaptive immune systems (31). In the context of infectious diseases, IL-17 initiates innate repair responses and host defense against bacterial infections at the mucosa, but, intriguingly, upregulated production of IL-17 can also exacerbate the severity of some inflammation (32). Interest in the efficacy and safety of novel therapeutic strategies based around IL-17 dramatically increases (33). IL-17 family consists of six members: IL-17A, IL-17B, IL-17C, IL-17D, IL-17E (also called IL-25), and IL-17F (34). It is well known that IL-17A and IL-17F from T helper17 cells induce pro-inflammatory chemokines, cytokines, and antimicrobial peptides, such as calprotectin, β-defensin, and cathelicidins (35). IL-17B and IL-17F participate in the immune response to protect pigs against F4^+^ ETEC infection and could aid in the design of future ETEC vaccines (36). IL-17C and its receptor IL-17RE are preferentially expressed in IECs and can be directly induced by bacteria (37). IL-17C can also trigger the production of inflammatory mediators, tight junctions, chemokines, and antimicrobial peptides, protecting host defense against microbial infection (38). LPS activation of TLR4 or flagellin activation of TLR5 can induce IL-17C production in small IECs (39, 40). Recently, Luo et al. reported that TLR5-mediated IL-17C expression in IECs enhances pig epithelial defense against F4^+^ ETEC infection by inducing the expression of antimicrobial peptides and tight junctions (41). In this study, *NFKBIA, AP-1* (*FOS* and *FOSB*), pro-inflammatory cytokine *IL-6*, chemokine *CXCL2, CXCL8*, and *CCL20* were directly related to the IL-17 pathway. In the peripheral blood leukocytes, the recombinant IL-17C could activate the NF-κB signaling and strongly promote the expression of chemokines (CXCL8, CXCL12, and CXCL13), pro-inflammatory factors (TNF-α, IL-1β, IL-6, and IFN-γ), and antibacterial peptide hepcidin (42). RNA-seq showed that the IL-17 pathway is enriched in the chicken lungs co-infected with *Mycoplasma gallisepticum* and ETEC, and IL-17 and some inflammasome-related genes (CXCL1, CXCL2, CXCL8, NF-κB, and AP-1) are significantly upregulated (43). Our data illustrate the importance of the IL-17 pathway in the pathogenesis of ETEC.

Furthermore, of the 131 DEGs, a total of 30 DEGs were involved in the immune response-associated KEGG pathways discussed earlier. These 30 “core DEGs” determined the IPEC-J2 cell immune response to ETEC infection. Correlation analysis of gene expression results showed that some genes in the “core DEGs,” such as *MAPK15, IL-6, CXCL2, NFKBIA, FOS*, and *TNFAIP3*, had the strongest correlation with other genes, indicating that the “core DEGs” had a coordinating function in the J2 cell response to ETEC infection. *IL-6* was identified as a differential node protein exerting the highest degree in the PPI network, which was likely to act as a key regulator in ETEC-mediated immune response in J2 cells. Besides, *FOS, MAPK15, JUNB, EDN1, DUSP1*, and the gene with unknown function (gene id: ENSSSCG00000031912) belonging to the 30 “core DEGs” had the highest scores for betweenness centrality, indicating they were most important for connections with other proteins. The FOS gene family consists of four members: FOS, FOSB, FOSL1, and FOSL2. These genes could encode leucine zipper proteins that can dimerize with proteins of the JUN family, thereby forming the transcription factor complex AP-1. Genes for AP-1 are immediate-early genes that regulate a wide range of physiological responses such as cell death, inflammation, and proliferation. In this study, FOS, FOSB, and JUNB genes were significantly upregulated and directly related to multiple enriched pathways, such as TLR, NF-κB, IL-17, MAPK, and TNF pathways, as shown by Venn analysis. Further qRT-PCR analysis validated the RNA-seq data. Our findings indicate that immune response-related signaling pathways mediated by nuclear transcription factor AP-1 and NF-κB determine the fate of ETEC-infected J2 cells and may also be the targets for further prevention and control.

The main strategies for preventing and controlling intestinal infection involve the overuse and misuse of antibiotics, which leads to the development of antibiotic resistance to commensal and opportunistic bacteria in both animals and humans. With China's ban on the addition of non-therapeutic antibiotics to animal feeds since January 1, 2020, the development of alternatives to conventional antibiotics is urgent. Probiotics are defined as “live microorganisms, which when administered in adequate amounts confer a health benefit to the host;” therefore, they represent a promising alternative to antibiotics for controlling enteric infections. Probiotic *Lactobacillus* is a major component of the gut microbiota and can protect the host against enteric pathogens through modulation of both local and systemic host immune responses (44, 45). In this study, we explored the regulatory effect of *L. reuteri* on the targeted genes and immune-related pathways obtained by RNA-seq. qRT-PCR resulted showed that ETEC infection significantly increased the expression of *CCL20, CXCL2, CXCL8, AP-1, IL-1, IL-6, IL-11, NFKBIA, MAP3K12, MAPK15, PTGS2*, and *TNFAIP3*, which pretreatment with *L. reuteri* inhibited these increases. *Lactobacillus reuteri* also attenuated the production of IL-6 induced by ETEC in the supernatant of J2 cells. The previous study using microarray analysis reported that *L. jensenii* TL2937 exerts anti-inflammatory immunobiotics for the prevention of inflammatory intestinal disorders in pigs by inhibiting the ETEC-induced high expression of chemokines (*CCL8, CXCL5, CXCL9, CXCL10*, and *CXCL11*) (46). *Lactobacillus rhamnosus* ATCC 7469 protects IECs from ETEC-induced damage, partly through the anti-inflammatory response involving synergism between TLR2 and nucleotide-binding and oligomerization domain 1 (47). Our previous study also showed that *L. rhamnosus* GR-1 ameliorates *E. coli*-induced inflammation and cell damage *via* attenuation of NLR NLRP3 and NLRC4 inflammasome activation (48, 49). Swine-derived probiotic *Lactobacillus plantarum* modulates porcine intestinal endogenous host defense peptide synthesis through TLR2/MAPK/AP-1 signaling pathway (50). Our data suggest that immune response-related signaling pathways obtained by RNA-seq represent the targets for preventing and controlling enteric ETEC infection in pigs.

In conclusion, ETEC infection elicits a strong immune response of porcine IECs, which is a result of the cooperation of multiple signaling pathways, including TNF, IL-17, MAPK, NF-κB, NLR, CLR, TLR, and cytokine–cytokine receptor interaction signaling pathways. Some key node genes, especially the nuclear transcription factor AP-1 and NF-κB, participate in the immune response to ETEC infection through an integral signal cascade and can be target molecules for prevention and control of enteric infection by probiotics.

## Data Availability Statement

The raw transcriptome data have been deposited in the US National Center for Biotechnology Information Sequence Read Archive database under accession no. SRR13291685–SRR13291693.

## Ethics Statement

The animal study was reviewed and approved by the Animal Care and Use Committee of Beijing University of Agriculture.

## Author Contributions

QW, D-FC, Y-HZhu, and Y-HZhang: conceived and designed the experiments and prepared the manuscript. J-XL, X-YC, PC, Y-DW, T-JS, ML, and R-YX: performed the RNA extraction, qRT-PCR, and ELISA. QW, D-FC, Y-HZhu, and Y-HZhang: performed the analysis of data. All authors contributed to the article and approved the submitted version.

## Conflict of Interest

The authors declare that the research was conducted in the absence of any commercial or financial relationships that could be construed as a potential conflict of interest.

## Publisher's Note

All claims expressed in this article are solely those of the authors and do not necessarily represent those of their affiliated organizations, or those of the publisher, the editors and the reviewers. Any product that may be evaluated in this article, or claim that may be made by its manufacturer, is not guaranteed or endorsed by the publisher.

## Supplementary Material

Supplementary Figure 1 Saturation curve of sequencing of each sample. Each color line represented the saturation curve of gene expression at different expression levels in the sample.

Supplementary Figure 2 Classification of new transcripts. New transcripts were classified according to the overlapping relationship between spliced transcripts and known transcripts. The percentage of new transcripts was shown.

Supplementary Figure 3 Analysis of novel gene expression and functional annotation. **(A)** The number of genes annotated using KEGG, GO, Pfam, Swiss-Prot, COG, and NR datasets. **(B)** COG, **(C)** GO, and **(D)** KEGG classifications of novel genes were summarized.

Supplementary Table 1 Sequences of oligonucleotide primers used for real-time PCR, length of the respective PCR product, and gene accession number.

Supplementary Table 2 Statistics of filtered transcriptome data.

Supplementary Table 3 Statistics of comparing with reference genome.

Supplementary Table 4 Statistical table of functional annotation of genes and transcripts.

Supplementary Table 5 Annotation of DEGs between CON and LPS groups.

Supplementary Table 6 Expression of 30 “core DEGs” involved in inflammatory signaling pathways.
