# Supplementary figures and images for: Transcriptome Analysis Identifies Strategies Targeting Immune Response-Related Pathways to Control Enterotoxigenic Escherichia coli Infection in Porcine Intestinal Epithelial Cells
Source: Front Vet Sci. 2021 Aug 10;8:677897. doi: 10.3389/fvets.2021.677897 (PMC8383179; doi:10.3389/fvets.2021.677897)

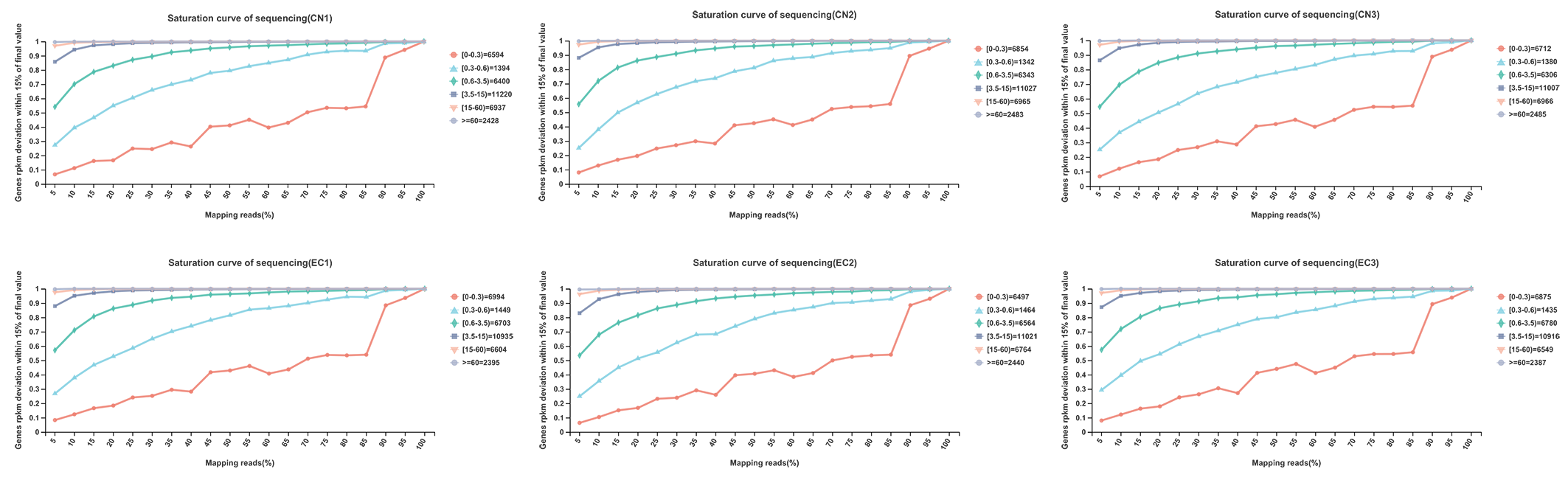

Supplement: Supplementary Figure 1 — Saturation curve of sequencing of each sample. Each color line represented the saturation curve of gene expression at different expression levels in the sample. [file Image_1.TIF]

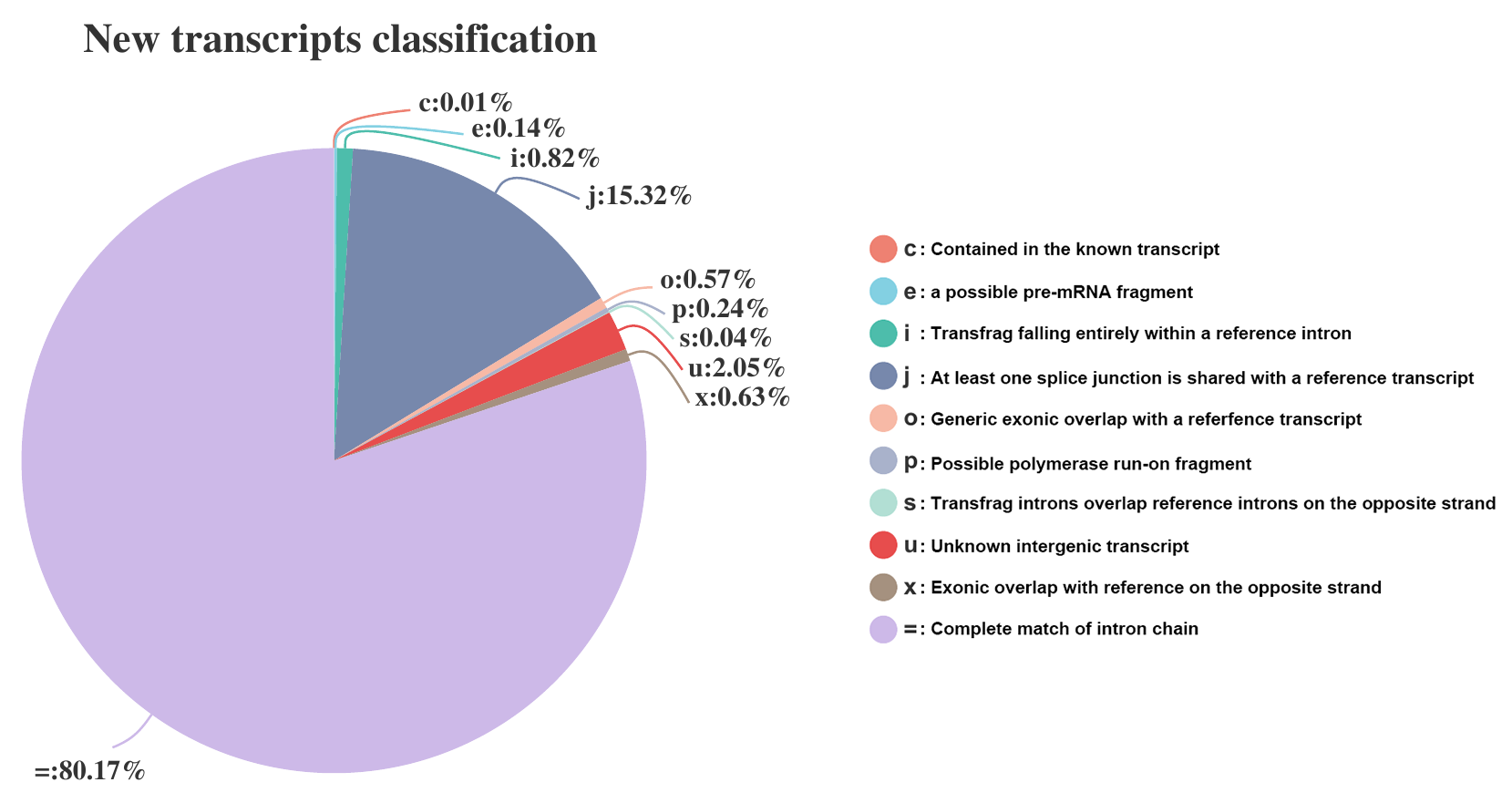

Supplement: Supplementary Figure 2 — Classification of new transcripts. New transcripts were classified according to the overlapping relationship between spliced transcripts and known transcripts. The percentage of new transcripts was shown. [file Image_2.TIF]

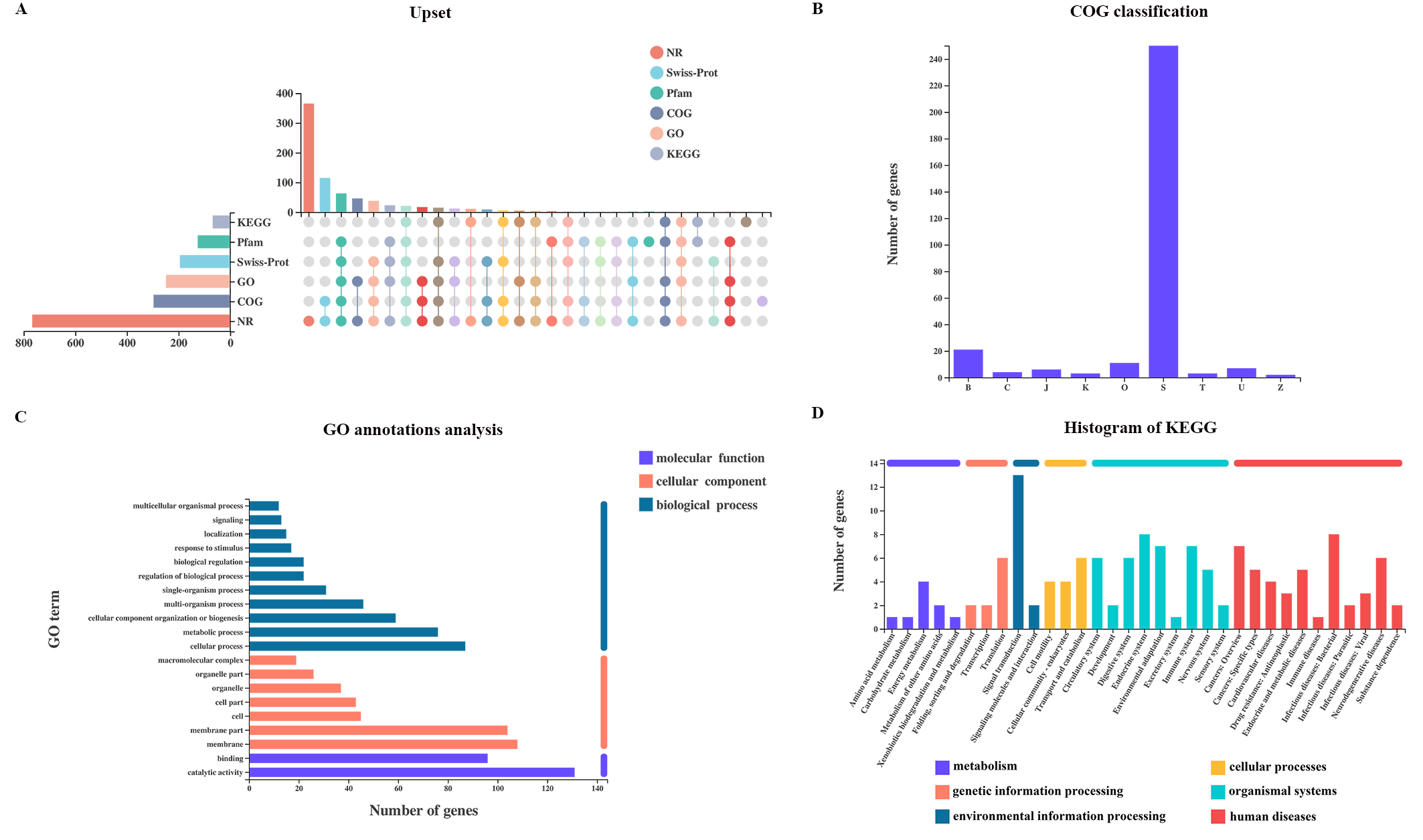

Supplement: Supplementary Figure 3 — Analysis of novel gene expression and functional annotation. (A) The number of genes annotated using KEGG, GO, Pfam, Swiss-Prot, COG, and NR datasets. (B) COG, (C) GO, and (D) KEGG classifications of novel genes were summarized. [file Image_3.TIF]
