# Supplementary material for: Transcriptome Analysis Identifies Strategies Targeting Immune Response-Related Pathways to Control Enterotoxigenic Escherichia coli Infection in Porcine Intestinal Epithelial Cells
Source: Front Vet Sci. 2021 Aug 10;8:677897. doi: 10.3389/fvets.2021.677897 (PMC8383179; doi:10.3389/fvets.2021.677897)
Supplement: Supplementary Table 1 — Sequences of oligonucleotide primers used for real-time PCR, length of the respective PCR product, and gene accession number. [file Table_1.docx]

**TABLES1** Sequences of oligonucleotide primers used for real-time PCR, length of the respective PCR product, and gene accession number

| **Gene product^a^** | **Primer** | | **Product size (bp)** | **Accession number** |
| --- | --- | --- | --- | --- |
|  | **Direction^b^** | **Sequence (5' to 3')** |  |  |
| *GADPH* | F | CCAGAACATCATCCCTGCTT | 229 | [NM_001206359](http://www.ncbi.nlm.nih.gov/entrez/viewer.fcgi?db=nucleotide&id=329744641) |
|  | R | GTCCTCAGTGTAGCCCAGGA |  |  |
| *CCL20* | F | GCTCCTGGCTGCTTTGATGTC | 143 | XM_005672261.2 |
|  | R | CATTGGCGAGCTGCTGTGTG |  |  |
| *CXCL2* | F  R | TGCAGACCGTGCAAGGAATT  TGGCTATGACTTCCGTTTGGT | 93 | NM_001001861 |
| *CXCL8* | F | CAGAACTTCGATGCCAGTGC | 174 | NM_213867 |
|  | R | CCTTCTGCACCCACTTTTCC |  |  |
| *IL-1β* | F | GCCAACGTGCAGTCTATGGAGTG | 91 | XM_021085847.1 |
|  | R | GGTGGAGAGCCTTCAGCATGTG |  |  |
| *IL-6* | F | GGCTGTGCAGATTAGTACC | 124 | AF518322 |
|  | R | CTGTGACTGCAGCTTATCC |  |  |
| *NFKBIA* | F | TGGTGTCGCTCTTGTTGAAGTGTG | 108 | NM_001005150.1 |
|  | R | GCTGCTGTATCCGAGTGCTTGG |  |  |
| *MAP3K12* | F | AGCACAAGCAGCAGCAGGAAG | 140 | XM_013986853.2 |
|  | R | CCGTACCTTCTTCACAGCCACTTC |  |  |
| *MAPK15* | F | TGAGTGGACACTAGAGGCGGATG | 87 | XM_021090469.1 |
|  | R | TGATAGAGGCGGCGGCGATAC |  |  |
| *FOS* | F | CTGTGGCTTCCCTTGATCTGAGTG | 139 | AJ132510 |
|  | R | TCCATGCTGCTGACGTTCTTGAC |  |  |
| *FOSB* | F | CCGGGCATGAGTGGCTACAG | 129 | AF120155 |
|  | R | CGTCTCCTCTCGGGGTCTCCT |  |  |
| *JUNB* | F | CTCAAGCTTGCCTCTTCGGA | 199 | XM_005674483.3 |
|  | R | GGTCGTCCAGGGCTTTTACA |  |  |
| *TNFAIP3* | F | ACAATGAGCAGGGGCGGAGAG | 150 | XM_021077624.1 |
|  | R | CTGAGCACTCGTGGCACAAGG |  |  |
