# Supplementary material for: Transcriptome Analysis Identifies Strategies Targeting Immune Response-Related Pathways to Control Enterotoxigenic Escherichia coli Infection in Porcine Intestinal Epithelial Cells
Source: Front Vet Sci. 2021 Aug 10;8:677897. doi: 10.3389/fvets.2021.677897 (PMC8383179; doi:10.3389/fvets.2021.677897)
Supplement: Supplementary Table 2 — Statistics of filtered transcriptome data. [file Table_2.DOCX]

**TABLE S2** Statistics of filtered transcriptome data

| Sample | Raw Reads | Clean Reads | Q20, % | Q30, % | GC content, % | Total mapped | Multiple mapped | Uniquely mapped |
| --- | --- | --- | --- | --- | --- | --- | --- | --- |
| CN1 | 55,788,312 | 55,106,030 | 98.19 | 94.72 | 51.09 | 53,165,424(96.48%) | 1,658,153(3.01%) | 51,507,271(93.47%) |
| CN2 | 62,509,150 | 61,780,380 | 98.31 | 95.04 | 51.03 | 59,633,404(96.52%) | 1,898,169(3.07%) | 57,735,235(93.45%) |
| CN3 | 56,802,686 | 56,132,040 | 98.28 | 94.95 | 51.06 | 54,166,748(96.5%) | 1,614,736(2.88%) | 52,552,012(93.62%) |
| EC1 | 61,642,826 | 60,924,650 | 98.28 | 94.96 | 51.28 | 58,746,213(96.42%) | 1,798,902(2.95%) | 56,947,311(93.47%) |
| EC2 | 51,258,814 | 50,643,548 | 98.25 | 94.88 | 51.44 | 48,855,176(96.47%) | 1,930,688(3.81%) | 46,924,488(92.66%) |
| EC3 | 60,157,528 | 59,437,822 | 98.29 | 94.98 | 51.35 | 57,248,737(96.32%) | 1,776,854(2.99%) | 55,471,883(93.33%) |

Clean Reads: reads of filtering out the linker and low-quality bases. Clean Base: the total number of bases filtered, ie the number of clean reads × length. Q30: bases with correct recognition rates above 99.9%. GC Content: number of G+Cs as a total number of bases percentage. Total mapped: the number of clean reads that can be mapped to the genome. Multiple mapped: the number of clean reads with multiple alignment positions on the reference sequence. Uniquely mapped: the number of clean reads with unique alignment positions on the reference sequence.
