# Supplementary material for: Transcriptome Analysis Identifies Strategies Targeting Immune Response-Related Pathways to Control Enterotoxigenic Escherichia coli Infection in Porcine Intestinal Epithelial Cells
Source: Front Vet Sci. 2021 Aug 10;8:677897. doi: 10.3389/fvets.2021.677897 (PMC8383179; doi:10.3389/fvets.2021.677897)
Supplement: Supplementary Table 3 — Statistics of comparing with reference genome. [file Table_3.DOCX]

**TABLE S3** Statistics of comparing with reference genome

| FPKM Inteval | CN1 | CN2 | CN3 | EC1 | EC2 | EC3 |
| --- | --- | --- | --- | --- | --- | --- |
| 0-0.3 | 6,594 | 6,854 | 6,712 | 6,994 | 6,497 | 6,875 |
| 0.3-0.6 | 1,394 | 1,342 | 1,380 | 1,449 | 1,464 | 1,435 |
| 0.6-3.5 | 6,400 | 6,343 | 6,306 | 6,703 | 6,564 | 6,780 |
| 3.5-15 | 11,220 | 11,027 | 11,007 | 10,935 | 11,021 | 10,916 |
| 15-60 | 6,937 | 6,965 | 6,966 | 6,604 | 6,764 | 6,549 |
| >60 | 2,428 | 2,483 | 2,485 | 2,395 | 2,440 | 2,387 |
