# Supplementary material for: Transcriptome Analysis Identifies Strategies Targeting Immune Response-Related Pathways to Control Enterotoxigenic Escherichia coli Infection in Porcine Intestinal Epithelial Cells
Source: Front Vet Sci. 2021 Aug 10;8:677897. doi: 10.3389/fvets.2021.677897 (PMC8383179; doi:10.3389/fvets.2021.677897)
Supplement: Supplementary Table 6 — Expression of 30 “core DEGs” involved in inflammatory signaling pathways. [file Table_6.DOCX]

**TABLE S6** Expression of 30 ‘core DEGs’ involved in inflammatory signaling pathways

| **Gene id** | **Gene_name** | **Gene description** | **Log2FC** | ***P* value** | **adjusted *P* value** |
| --- | --- | --- | --- | --- | --- |
| ENSSSCG00000008963 | AREG | amphiregulin | 1.04 | 5.36E-16 | 7.04E-14 |
| ENSSSCG00000027426 | BCL3 | BCL3, transcription coactivator | 1.38 | 3.63E-10 | 2.81E-08 |
| ENSSSCG00000016254 | CCL20 | C-C motif chemokine ligand 20 | 5.41 | 3.40E-15 | 4.10E-13 |
| ENSSSCG00000012915 | CLCF1 | cardiotrophin like cytokine factor 1 | 1.78 | 3.13E-72 | 1.83E-69 |
| ENSSSCG00000008959 | CXCL2 | chemokine (C-X-C motif) ligand 2 | 2.85 | 2.70E-49 | 9.26E-47 |
| ENSSSCG00000008953 | CXCL8 | C-X-C motif chemokine ligand 8 | 1.77 | 2.05E-77 | 1.41E-74 |
| ENSSSCG00000016991 | DUSP1 | dual specificity phosphatase 1 | 1.88 | 2.76E-07 | 1.32E-05 |
| ENSSSCG00000040815 | DUSP5 | dual specificity phosphatase 5 | 1.24 | 2.28E-53 | 9.19E-51 |
| ENSSSCG00000035598 | EDN1 | endothelin 1 | 1.46 | 1.68E-35 | 4.02E-33 |
| ENSSSCG00000003471 | EPHA2 | EPH receptor A2 | 1.03 | 1.45E-66 | 7.69E-64 |
| ENSSSCG00000002383 | FOS | Fos proto-oncogene, AP-1 transcription factor subunit | 3.30 | 3.19E-78 | 2.33E-75 |
| ENSSSCG00000031616 | FOSB | FosB proto-oncogene, AP-1 transcription factor subunit | 4.40 | 1.33E-60 | 6.24E-58 |
| ENSSSCG00000040725 | IL11 | interleukin 11 | 2.66 | 1.49E-09 | 1.06E-07 |
| ENSSSCG00000008090 | IL1A | interleukin 1 alpha | 1.23 | 1.22E-14 | 1.43E-12 |
| ENSSSCG00000020970 | IL6 | interleukin 6 | 1.35 | 8.34E-07 | 3.51E-05 |
| ENSSSCG00000013735 | JUNB | JunB proto-oncogene, AP-1 transcription factor subunit | 2.29 | 3.05E-38 | 8.28E-36 |
| ENSSSCG00000040961 | LIF | LIF, interleukin 6 family cytokine | 1.65 | 1.57E-08 | 9.29E-07 |
| ENSSSCG00000000275 | MAP3K12 | mitogen-activated protein kinase kinase kinase 12 | 1.08 | 2.55E-06 | 9.52E-05 |
| ENSSSCG00000035933 | MAPK15 | mitogen-activated protein kinase 15 | 1.61 | 1.80E-05 | 5.02E-04 |
| ENSSSCG00000001952 | NFKBIA | NFKB inhibitor alpha | 2.65 | 4.73E-91 | 3.95E-88 |
| ENSSSCG00000031321 | NR4A1 | nuclear receptor subfamily 4 group A member 1 | 4.65 | 0 | 0 |
| ENSSSCG00000010312 | PLAU | plasminogen activator, urokinase | 1 | 8.12E-18 | 1.12E-15 |
| ENSSSCG00000003928 | PLK3 | polo like kinase 3 | 1.43 | 1.59E-33 | 3.58E-31 |
| ENSSSCG00000015579 | PTGS2 | prostaglandin-endoperoxide synthase 2 | 2.18 | 2.35E-115 | 2.50E-112 |
| ENSSSCG00000015549 | RNASEL | ribonuclease L | −1.04 | 3.26E-03 | 3.25E-02 |
| ENSSSCG00000004154 | TNFAIP3 | TNF alpha induced protein 3 | 2.14 | 4.68E-39 | 1.37E-36 |
| ENSSSCG00000029160 |  | - | 1.80 | 2.51E-179 | 4.90E-176 |
| ENSSSCG00000031657 |  | JunD proto-oncogene, AP-1 transcription factor subunit | 1.59 | 1.53E-86 | 1.19E-83 |
| ENSSSCG00000031912 |  | - | 3.40 | 0 | 0 |
| ENSSSCG00000036956 |  | suppressor of cytokine signaling 3 | 1.57 | 5.54E-08 | 2.97E-06 |
